# Supplementary material for: Molecular insights into the catalytic mechanism of plasticizer degradation by a monoalkyl phthalate hydrolase
Source: Commun Chem. 2023 Mar 1;6:45. doi: 10.1038/s42004-023-00846-0 (PMC9977937; doi:10.1038/s42004-023-00846-0)
Supplement: Supplementary file 2 — Supplementary Information [file 42004_2023_846_MOESM2_ESM.pdf]

Molecular insights into the catalytic mechanism of plasticizer degradation by a monoalkyl phthalate hydrolase

Yebao Chen[1], Yongjin Wang[2], Yang Xu[3], Jiaojiao Sun[3], Liu Yang[3], Chenhao Feng[3], Jia Wang[3], Yang Zhou[2*], Zhi-Min Zhang[2*], Yonghua Wang[3, 4*]

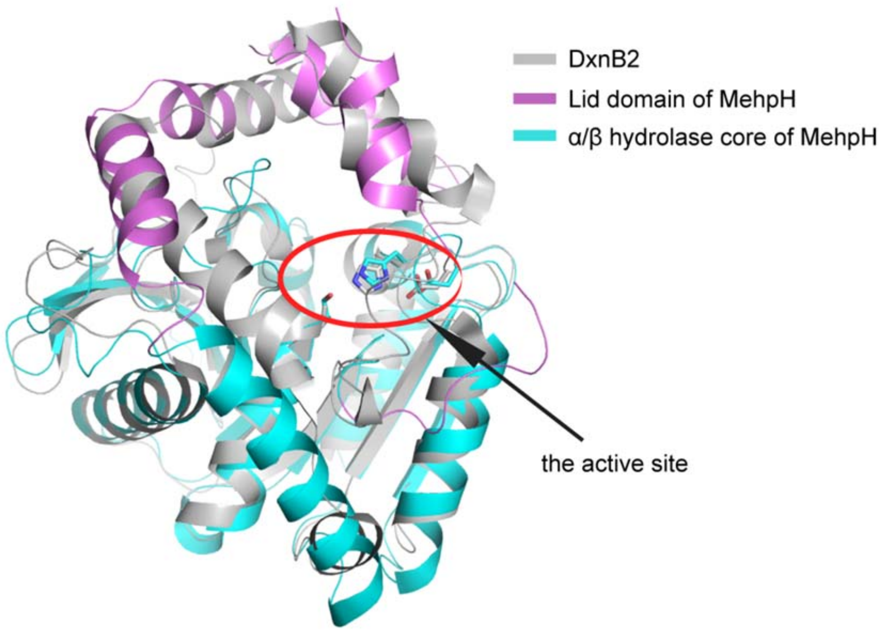

Supplementary figure 1. Structure superposition of MehpH and DxnB2. DxnB2 (PDB code: 4LXH) is shown in grey. MehpH is colored in the same scheme as in figure 1. The triads in the active site of MehpH and DxnB2 are shown as sticks.

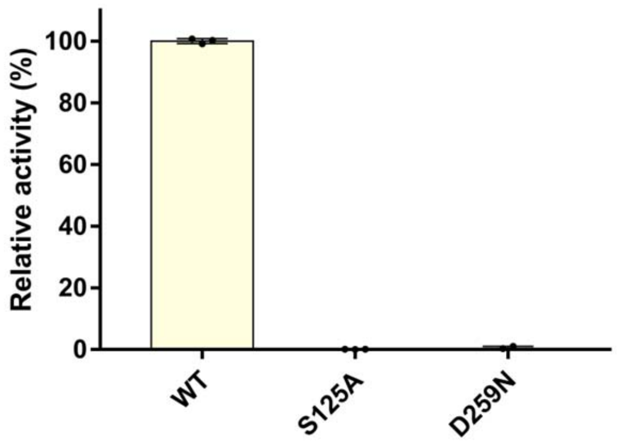

Supplementary figure 2. *In vitro* enzymatic assay of MehpH D259N. Error bars represent the s.d. values obtained in triplicate experiments.

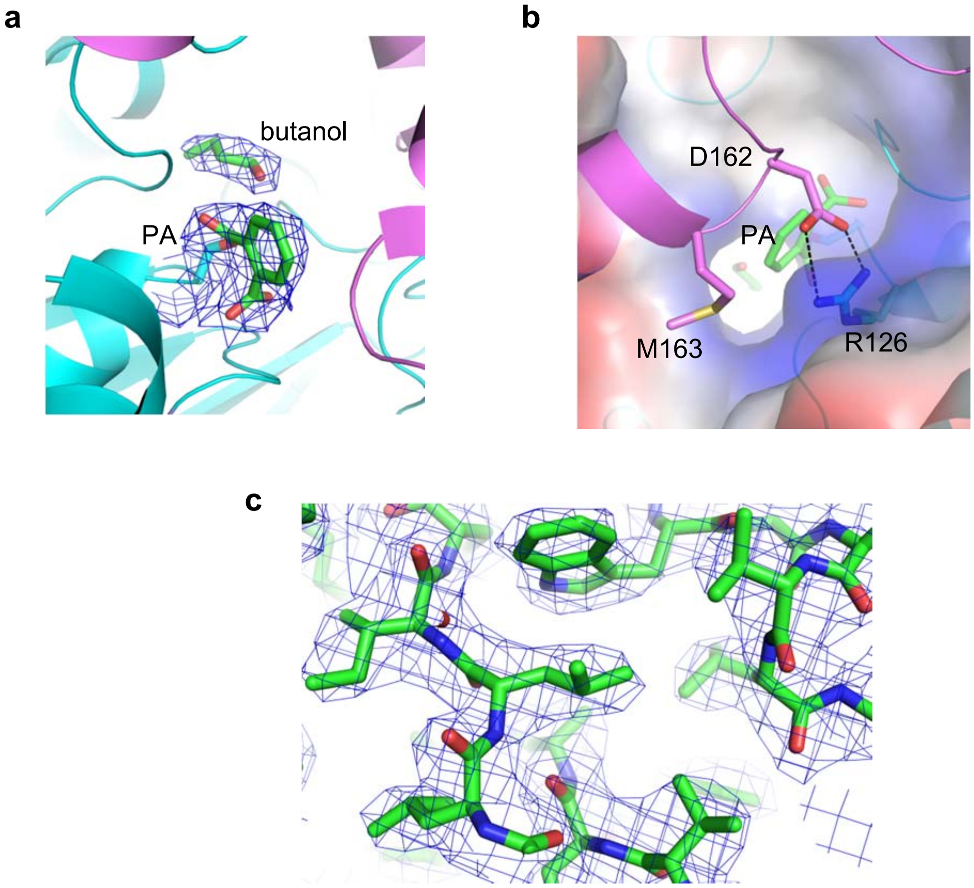

Supplementary figure 3. The active site of MehpH. (a) 2Fo-Fc omit map of PA and butanol in the structure of MehpH-ligand complex at a contour level of 1.0 σ. The protein is colored in the same scheme as shown in figure 1. PA and butanol are shown as sticks. **(b)** The binding pocket of PA and butanol is covered by M163 and D162 from the NC loop. The hydrogen bonds are depicted as black dashed lines. **(c)** 2Fo-Fc omit map of portion of the apo-MehpH structure at a contour level of 1.0 σ.

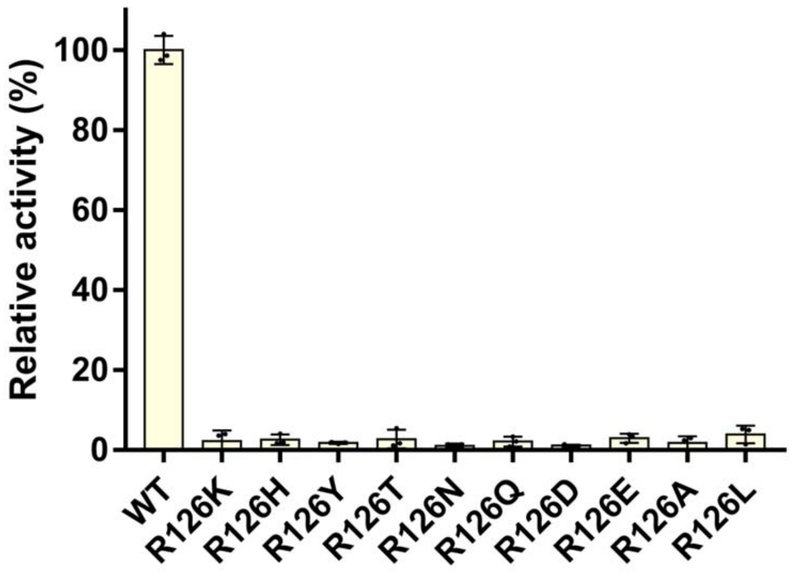

Supplementary figure 4. ***In vitro*** **enzymatic assay of MehpH with R126 replaced by different amino acids.** Error bars represent the s.d. values obtained in triplicate experiments.

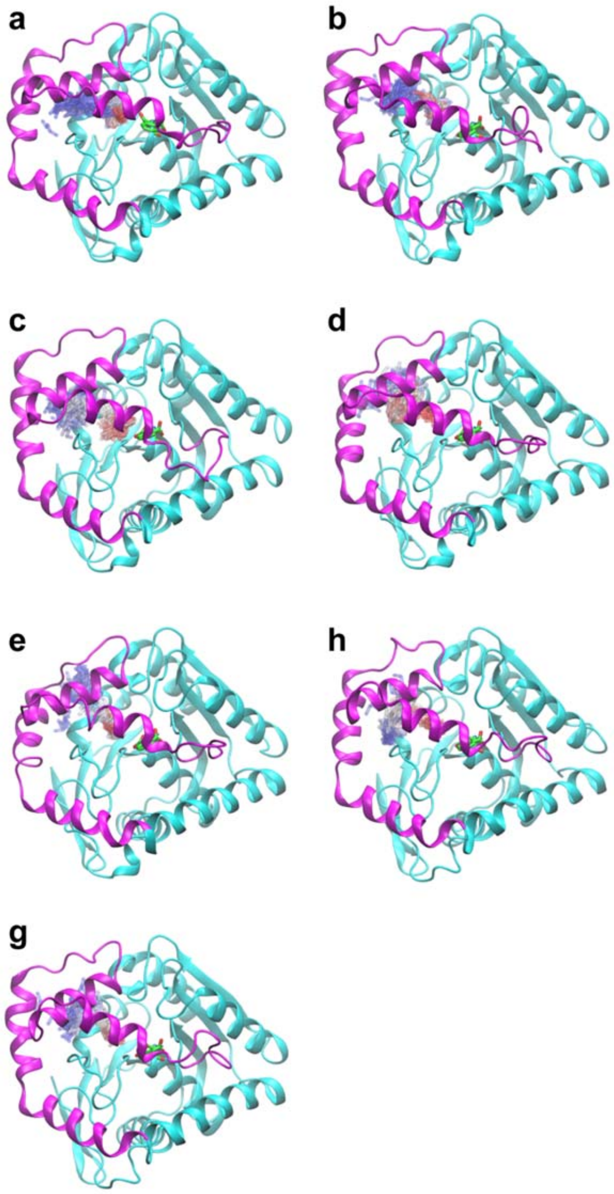

Supplementary figure 5. Unbinding process of butanol from independent MD simulation runs. Time evolution of butanol is colored from red (t=0) to blue (unbound).

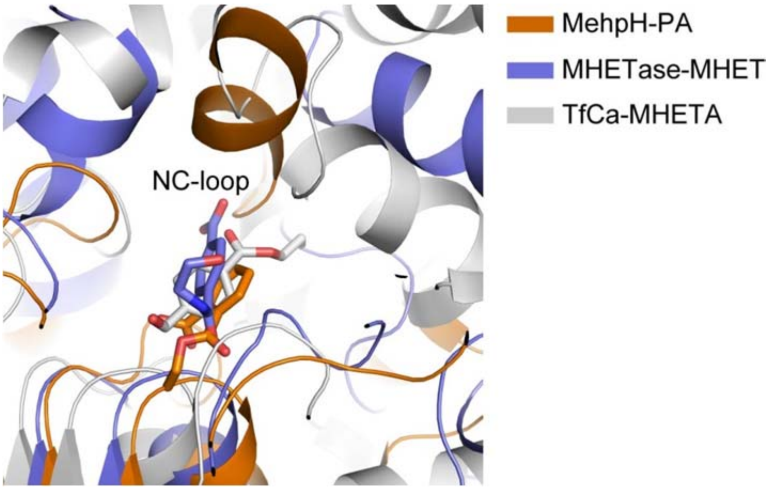

Supplementary figure 6. Comparison of the active sites in MehpH and PET-degrading enzymes. The structure of MehpH-PA complex is colored in brown. The structure of MHETases in complex with MHET (PDB code: 6JTT) is colored in blue. The structure of TfCa-MHETA complex (PDB code: 7W1J) is colored in grey.
