# Supplementary material for: Molecular insights into the catalytic mechanism of plasticizer degradation by a monoalkyl phthalate hydrolase
Source: Commun Chem. 2023 Mar 1;6:45. doi: 10.1038/s42004-023-00846-0 (PMC9977937; doi:10.1038/s42004-023-00846-0)
Supplement: Supplementary file 3 — Description of Additional Supplementary Files [file 42004_2023_846_MOESM3_ESM.pdf]

Description of Additional Supplementary Files

File name: Supplementary Data 1

Description: The coordination of the structure of MehpH.

File name: Supplementary Data 2

Description: The coordination of the structure of MehpH in complex with ligands.

File name: Supplementary Data 3

Description: The representative structures for metastable state M1.

File name: Supplementary Data 4

Description: The representative structures for metastable state M2.
